# Investigating the neuroprotective effect of heparin in improving mitochondrial function in rats with cardiac arrest cardiopulmonary resuscitation based on transcriptome sequencing

**DOI:** 10.3389/fmed.2025.1712265

**Published:** 2026-01-08

**Authors:** Biyun Tian, Xin Liu, Fa Wang, Yang Gu, Xiaohong Zhou, Ningkang Li, Qingshan Ye, Yan Li

**Affiliations:** 1 Ningxia Medical University, Yinchuan City, China; 2 Department of Anesthesiology, People's Hospital of Ningxia Hui Autonomous Region, Yinchuan City, China; 3 Department of Anesthesiology, Bayannur Hospital, Bayannur City, China

**Keywords:** neuroprotective effect, heparin, mitochondrial function, cardiac arrest, cardiopulmonary resuscitation, transcriptome sequencing

## Abstract

**Background:**

Cardiac arrest (CA) and subsequent cardiopulmonary resuscitation (CPR) often lead to severe brain injury, primarily driven by mitochondrial dysfunction and ischemia–reperfusion injury. While heparin is a known anticoagulant, its potential neuroprotective role through mitochondrial regulation post cardiac arrest and cardiopulmonary resuscitation (CA-CPR) remains poorly understood. This study aimed to explore the brain-protective mechanisms of heparin, focusing on its effects on mitochondrial function using transcriptome sequencing in a rat model of asphyxial CA-CPR.

**Methods:**

Male Sprague–Dawley rats were randomized into three groups: Sham, CPR (Model), and CPR + Heparin (CPR + HP, 0.5 mg/kg IV upon CPR initiation). Neurological function was assessed using modified Neurological Severity Scores (mNSS) over 7 days. Hippocampal tissues were collected for transcriptome sequencing, qPCR validation, histological examination (HE staining), transmission electron microscopy (TEM), and biochemical assays (ATP and ROS levels). Bioinformatic analyses included differential gene expression, KEGG/GO enrichment, protein–protein interaction (PPI) networks, and ROC analysis.

**Results:**

Heparin treatment significantly improved neurological outcomes, reduced cerebral edema, and enhanced the 20-day survival rate (55% vs. 30% in CPR group, *p* < 0.05). Transcriptome analysis identified 757 differentially expressed genes (DEGs) between CPR and CPR + HP groups. Enrichment analysis revealed significant involvement of mitochondrial-related pathways, complement/coagulation cascades, and the AGE-RAGE signaling pathway. Intersection with a mitochondrial gene database identified three hub genes—Epsa1, Idh2, and Hif3a,which were significantly downregulated in the CPR group but restored by heparin treatment (*p* < 0.05). ROC analysis confirmed their diagnostic value (AUC > 0.75). Heparin also increased ATP content, reduced ROS levels, and preserved neuronal and mitochondrial ultrastructure.

**Conclusion:**

Heparin provided neuroprotection after CA-CPR by ameliorating mitochondrial dysfunction via multi-target regulation of key genes (Epsa1, Idh2, and Hif3a), enhancing energy metabolism, reducing oxidative stress, and inhibiting hyperactivated coagulation-inflammation cascades. These findings highlight heparin’s potential as an adjunctive therapy for improving neurological outcomes post-resuscitation.

## 1 Introduction

Cardiac arrest (CA) is one of the most critical emergencies in the field of emergency medicine, accounting for 80% to 85% of all sudden deaths (1–3). It is characterized by sudden onset, a narrow rescue window, and extremely high rates of disability and mortality. Patients often present with sudden loss of consciousness, pulselessness, and respiratory arrest. If effective cardiopulmonary resuscitation (CPR) is not initiated within the “Golden Four Minutes,” it can rapidly progress to irreversible brain ischemia and hypoxia damage or even death (4–6). Despite continuous advancements in modern advanced life support technologies, the rate of return of spontaneous circulation (ROSC) has improved, but the discharge survival rate and the rate of good neurological function remain unsatisfactory (7, 8). The key bottleneck lies in the multiple organ failure caused by post-cardiac arrest syndrome (PCAS), especially the ischemia–reperfusion injury-induced heart and brain damage (9).

Research indicates that the imbalance between the structural integrity and functional homeostasis of mitochondria is one of the core pathological links in heart and brain injury following cardiac arrest and cardiopulmonary resuscitation (10). As the “powerhouses” of energy metabolism in cardiomyocytes and neurons, mitochondria are rich in tricarboxylic acid cycle enzymes, respiratory chain complexes, and ATP synthase. They generate ATP through oxidative phosphorylation, regulate intracellular calcium homeostasis and reactive oxygen species (ROS) levels, and maintain the excitation-contraction coupling of cardiomyocytes and the steady state of neuronal electrical activity (11, 12). During CA, the interruption of systemic circulation leads to ischemia and hypoxia, sharply reducing mitochondrial ATP production, decreasing Na^+^/K^+^-ATPase activity, and causing membrane depolarization and calcium overload (13, 14). In the reperfusion stage, after blood flow is restored, the electron leakage in the mitochondrial respiratory chain increases, leading to a burst release of ROS (15, 16), which triggers oxidative stress, persistent opening of the mitochondrial permeability transition pore, and further induces cardiomyocyte apoptosis and neuronal excitotoxic injury. Additionally, the release of mitochondrial DNA and formyl peptides and other damage-associated molecular patterns from damaged mitochondria activates systemic inflammatory responses, exacerbating microcirculatory disorders and blood–brain barrier disruption (17–19). Therefore, targeting the protection of mitochondrial function has become a key strategy for improving the quality of heart and brain resuscitation after CA-CPR.

The latest research has confirmed that heparin treatment (including low-dose heparin anticoagulation during the resuscitation period or early anticoagulation intervention after resuscitation) can indirectly improve mitochondrial function by inhibiting the coagulation cascade reaction, reducing microthrombus formation and inflammatory response, significantly alleviating myocardial depression and neuronal apoptosis after CA-CPR, and improving long-term cardiac function and neurological cognitive prognosis (20–22). However, the molecular network of mitochondrial damage in the CA-CPR scenario and the potential targets regulated by heparin treatment have not been systematically clarified yet. Given that transcriptome sequencing can comprehensively analyze the gene expression profile characteristics of ischemia–reperfusion injury, this study intends to adopt this technology to reveal the mechanism of mitochondria in cardiac and cerebral injury after CA-CPR at the level of key signaling pathways and regulatory networks, and clarify the scientific connotation of the multi-dimensional intervention of “anticoagulation-anti-inflammation-mitochondrial protection” by heparin treatment, providing a theoretical basis for subsequent mechanism verification at the cellular and animal levels.

## 2 Materials and methods

### 2.1 Animals

This experiment was approved by the Ethics Committee of Ningxia Medical University Affiliated Ningxia Hui Autonomous Region Region People’s Hospital (Approval Number: [2023]-NZR-093). The animal center of Ningxia Medical University provided 112 male Sprague–Dawley rats, weighing 250–300 g. The rats were housed in Specific Pathogen Free (SPF) environment and had free access to food and water. The room temperature was maintained at 18–24 °C, and the humidity was controlled at 40% to 50%. A 12-h/12-h day-night cycle was implemented.

### 2.2 Ethics statement

The study was conducted strictly in accordance with the Measures for the Management of Laboratory Animals in the Ningxia Hui Autonomous Region [issued by the (Science and Technology Department of Ningxia Hui Autonomous Region, China, in 2021, No. 19)]. All efforts were made to minimize suffering Anesthesia was administered using continuous inhalation of isoflurane (2%–5%) throughout the experimental procedure. Prior to making the surgical incision and before suturing the incision, 0.1 mL of 1% lidocaine was injected locally for wound infiltration to provide immediate relief from local pain.

### 2.3 Humane endpoints

Humane endpoints were established for each animal. The specific endpoint criterion was loss of consciousness and pain sensation accompanied by muscle relaxation, assessed beginning 2 h after the return of spontaneous circulation (ROSC). Once an animal fulfilled this criterion, euthanasia was conducted within 10 to 12 min though continuous inhalation of 5% isoflurane to ensure immediate loss of consciousness and death. The rats that never achieved return of spontaneous circulation (ROSC) or developed severe ischemia–reperfusion injury 1 to 3 days post-ROSC were euthanized at the end of the experiments.

No animals were found dead prior to meeting the endpoint criteria. On the first day post-modeling, heart rate, blood pressure, respiration, end-expiratory CO₂ and body temperature were recorded within the first 2 h. General health and behavior were checked daily thereafter. All personnel involved in animal handling and euthanasia received professional training and are certified for laboratory animal work.

### 2.4 Experimental grouping

**Figure 1:**
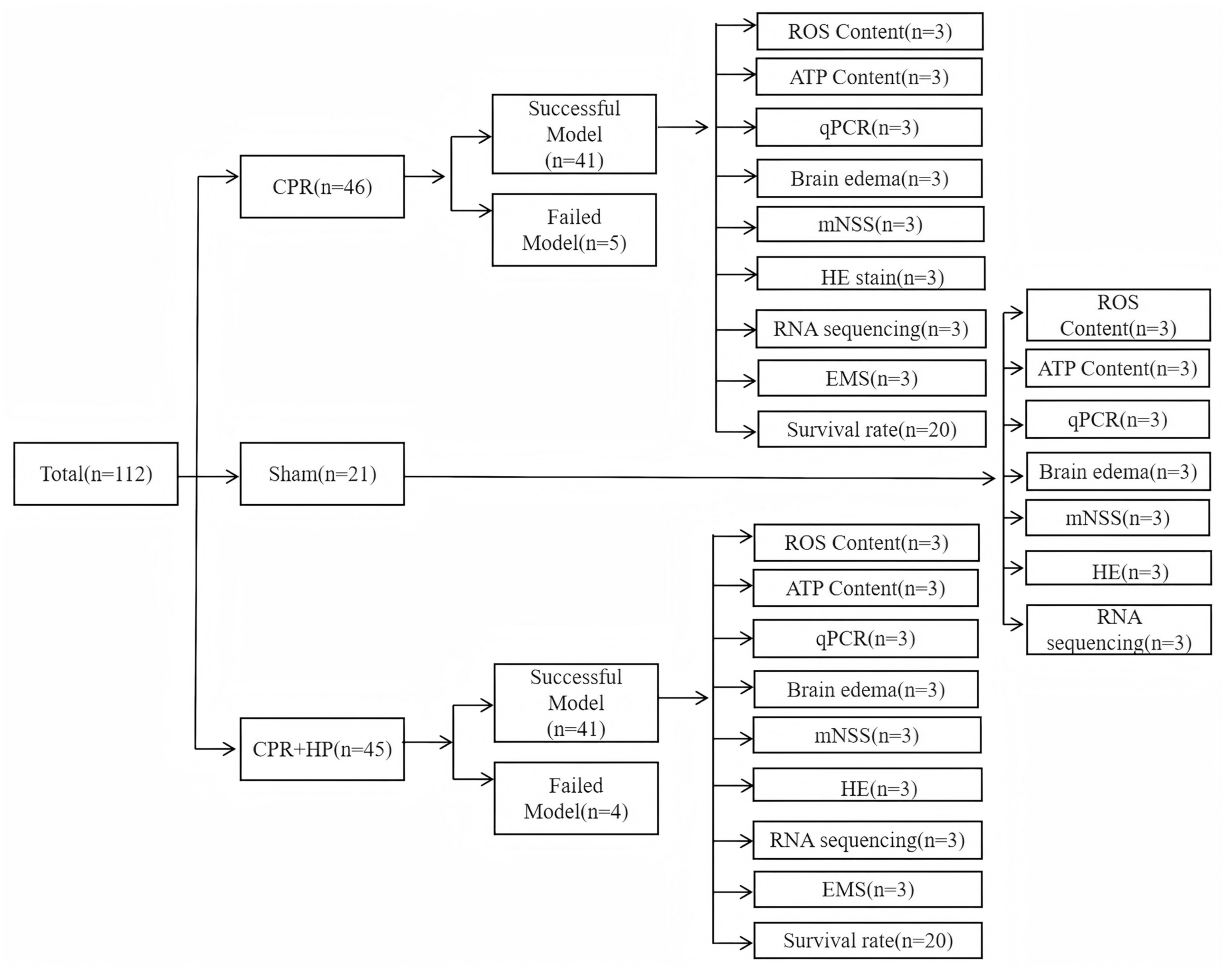
Summarizes the overall experimental design, animal grouping, and sample sizes for various endpoint measurements. A total of 112 rats were randomized into three groups: Sham (n = 21), CPR (n = 46 attempted, 41 successful), and CPR + HP (n = 45 attempted, 41 successful). The sample sizes for each experimental endpoint are indicated. Specifically, a subset of animals that completed the mNSS assessments were subsequently used for HE staining. The figure also details the sample allocation for survival analysis (n = 20 per group for CPR and CPR + HP), other biochemical assays (ATP, ROS), molecular analyses (qPCR, RNA sequencing), and histological examinations (electron microscopy).

The animals were divided into three groups using a random number method. The details were shown in Figure 1:

(1) Sham Group (n = 21): Sham operation group. After the rats were anesthetized, tracheal intubation and vascular puncture catheterization were performed, but no CA-CPR operation was carried out.
(2) CPR Group (n = 46): Cardiopulmonary resuscitation group. After the rats were anesthetized, tracheal intubation and vascular puncture catheterization were performed, and CA-CPR operation was carried out. At the moment of CPR, an equal dose of normal saline was intravenously injected.
(3) CPR + HP Group (n = 45): Cardiopulmonary resuscitation with heparin treatment group. After the rats were anesthetized, tracheal intubation and vascular puncture catheterization were performed, CA-CPR operation was carried out, and heparin 0.5 mg/Kg was intravenously injected at the onset of CPR.

### 2.5 Experimental equipment, drugs, reagents and consumables

Small animal anesthesia machine (Shanghai Yuran Scientific Instrument Co., ABS single-channel); Video laryngoscope (Shenzhen Taishilang Technology Co., MS500); Multi-channel biological signal acquisition instrument (Anhui Zhenghua Biological Instrument Equipment Co., MD-3000D); Small animal ventilator (Anhui Zhenghua Biological Instrument Equipment Co., DW-3000H); Oxygen generator (Jiangsu Yujoy Medical Equipment Co., YU300); End-expiratory carbon dioxide monitor (Beijing Jinjiaxin Trading Co., KMI605C); Small animal electronic rectal thermometer (Suzhou Zhongluotong Testing Instrument Co., TH212); Square box (Shanghai Xinsong Information Technology Co.); Fully automatic tissue embedding machine (Germany, Leica); Pathological cryostat (Shenzhen Ruiwode Life Science Technology Co., Ltd.); Optical microscope (Japan, Olympus); Electric heating air drying oven (Shanghai Shengxin Scientific Instrument Co., 101AS-3); Ultra-clean workbench (Suzhou Purification Equipment Co., SW-CJ-1FD); High-speed freezing centrifuge (USA, Thermo, Sorvall ST 16R); Centrifuge (Germany, EPPendorf, 5430); Analytical balance (Germany, Sartorius, BL310/BL21S); Constant temperature shaker (Shanghai Heng Science Instrument Co., THZ-98A); Ultra-low temperature storage box (Qingdao Baimi Instrument Co., DW-86BL980D); Refrigerator (Qingdao Baimi Instrument Co., BYC-600G); Pipette (Germany, EPPendorf, 10/200/300/1,000 μL); Multi-functional enzyme analyzer (Switzerland, TECAN, Spark); Transmission electron microscope (Japan, Hitachi, HT7800); Fully automatic tissue embedding machine (Germany, Leica); Pathological cryostat (China, Shenzhen Ruiwode Life Science Technology Co., Ltd.); 4% paraformaldehyde fixative (China, Shanghai Byungyoun Bio-technology Co., Ltd.); Safranin (China, Shanghai Byungyoun Bio-technology Co., Ltd.); Reverse transcription kit (Wuhan Saiwei Biotechnology Co., Ltd., G3337), Animal RNA extraction kit (MagBeads, T102096).

Isoflurane (Shenzhen Ruiwode Life Science Technology Co., Ltd.); Lidocaine injection (Shanghai Chaohui Pharmaceutical Co., Ltd.); Heparin sodium injection (Guojun Group Rensheng Pharmaceutical Co., Ltd.); Sulfonated atracurium besylate injection (Nanjing Jianyou Biochemical Pharmaceutical Co., Ltd.); Hydrochloric acid epinephrine injection (Yuan Da Medicine Co., Ltd.); 4% paraformaldehyde fixative (Shanghai Byungyoun Bio-technology Co., Ltd.); Safranin (Shanghai Byungyoun Bio-technology Co., Ltd.); Phosphate buffer solution (PBS) (Wuhan Ponus Life Science Technology Co., Ltd.); Anhydrous ethanol (Shanghai Byungyoun Bio-technology Co., Ltd.); DPX mounting agent (Shanghai Mao Kang Bio-technology Co., Ltd.)

14G cannula - tracheal tube (USA, Insyte BD Medical); 24G cannula needle (Shanghai Bide Medical Devices Co., 383083-Y); Infusion three-way connector (Shanghai Bide Medical Devices Co., 394601); 96-well plate (Guangzhou Jieti Bio-filtration Co., Ltd., TCP011096); Centrifuge tube (Guangzhou Jieti Bio-filtration Co., Ltd., CFT411150).

### 2.6 Establishment of a rat model of asphyxial cardiac arrest and cardiopulmonary resuscitation

**Figure 2:**
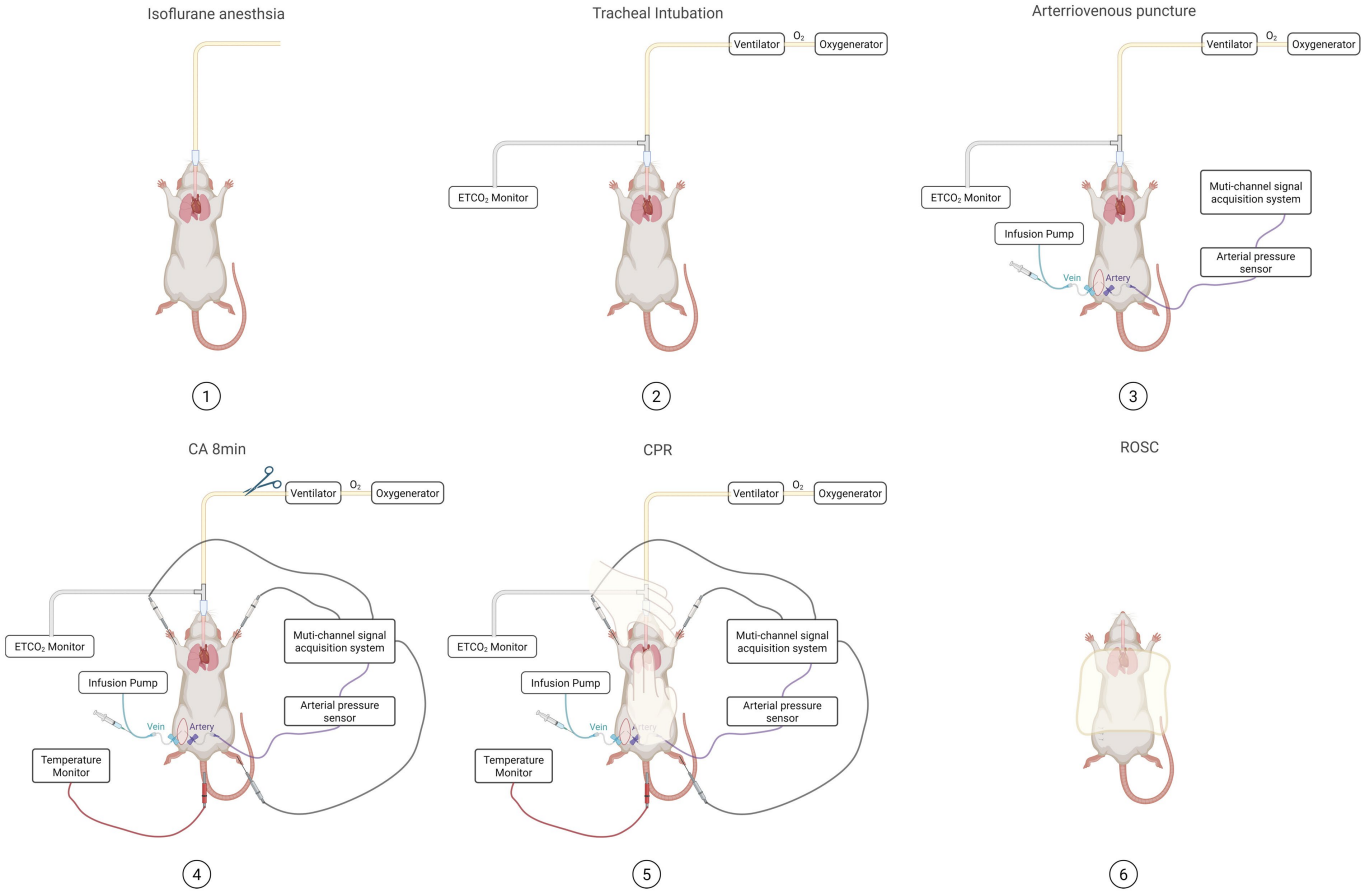
Illustrates the detailed procedural setup and sequential steps for establishing the asphyxial cardiac arrest-cardiopulmonary resuscitation (CA-CPR) model in rats. The figure depicts the six critical phases: (1) Isoflurane anesthesia; (2) Tracheal intubation with ventilator connection;(3)Arteriovenous puncture for hemodynamic monitoring and drug administration; (4) 8-min asphyxia period to induce cardiac arrest (MAP ≤ 20 mmHg); (5) Initiation of cardiopulmonary resuscitation with mechanical ventilation and chest compressions; (6) Achievement of return of spontaneous circulation (ROSC). Key equipment including the ventilator, oxygenator, ETCO₂ monitor, multi-channel signal acquisition system, and infusion pump are indicated throughout the procedure.

**Figure 3:**
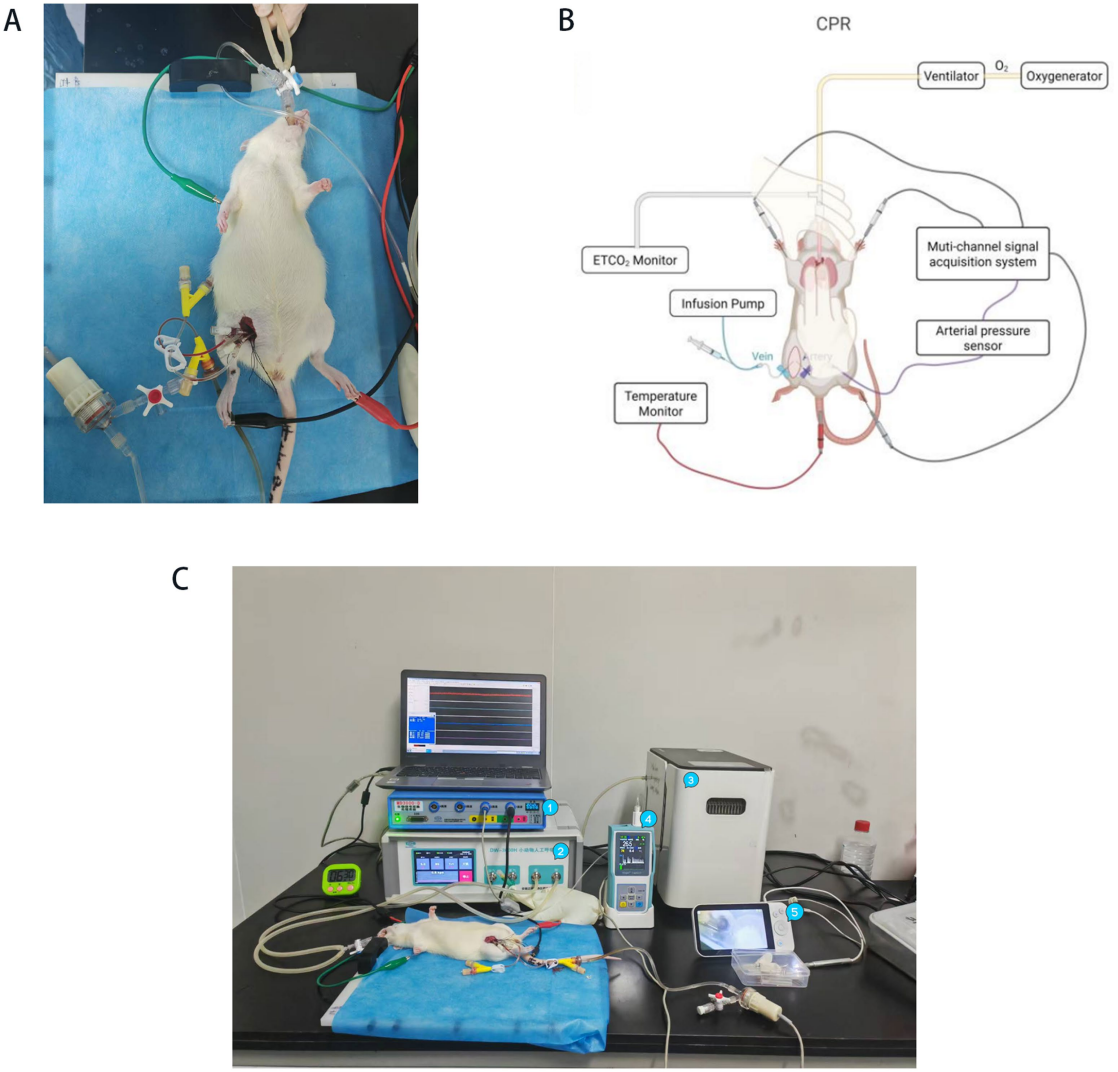
Schematic diagram of the cardiopulmonary resuscitation model for asphyxial cardiac arrest in rats. **(A)** Photograph of the rat model. **(B)** Overall schematic diagram of the model. (**C**) The equipment used for model construction includes: ① multi-channel signal acquisition system; ② small animal ventilator; ③ oxygen generator; ④ ETCO_2_ monitor; ⑤ video laryngoscope.

In this study, the asphyxial cardiac arrest and cardiopulmonary resuscitation (CA-CPR) method was used for modeling, and the schematic diagram is shown (Figures 2, 3).

### 2.7 Model preparation process

(1) Anesthesia, tracheal intubation and venous access: The rats were fasted for 12 h before the surgery, without water restriction. After being weighed, they were placed in the anesthesia box. 4%–5% isoflurane was inhaled to induce anesthesia for 3 min, followed by a supine position and fixation of the limbs on the operating table. Using an oral visual laryngoscope, a 14G cannula was inserted via the guiding guide wire, to a depth of 4 cm to 5 cm. After successful intubation, the end-tidal carbon dioxide partial pressure (ETCO_2_) monitor, ventilator, and anesthesia machine pipelines were connected. Anesthesia was maintained with 2% isoflurane inhaled. The oxygen supply machine was connected to the ventilator to maintain the inhaled oxygen concentration (Fraction Of Inspired O_2_, FiO_2_) at 1.0. The mechanical ventilation mode was not activated at the moment, and the rats’ spontaneous breathing was preserved. The right inguinal area of the rat was shaved and disinfected. A 1% lidocaine solution was locally infiltrated to anesthetize the skin and subcutaneous tissue of the surgical area, and the femoral arteries and veins were gradually dissected and exposed layer by layer, followed by insertion into 24G cannulas. After successful cannulation, the femoral artery was connected to the biological signal acquisition instrument’s arterial pressure sensor to monitor arterial blood pressure, and the femoral vein was connected to the infusion pump connection pipeline to continuously infuse normal saline at a rate of 2 mL/h.
(2) Asphyxia-induced cardiac arrest: The electrocardiogram (ECG) and rectal temperature of the rats were continuously monitored. A bolus intravenous injection of 0.2 mg/100 g cisatracurium was delivered via the femoral vein. Upon cessation of spontaneous breathing, mechanical ventilation was initiated on the ventilator with settings of a tidal volume of 10 mL/kg, a respiratory rate of 80 breaths per minute, and an inspiratory-to-expiratory ratio of 1:1. The respiratory rate was adjusted based on the ETCO_2_ monitor readings to maintain ETCO_2_ between 30 and 40 mmHg. The vital signs of the rats were continuously recorded. After a stable baseline was achieved for 5 min, mechanical ventilation was paused and the tracheal tube was removed. Timing began at 8 min. After the onset of asphyxia, the rat’s pupils changed from red to white, and the skin and mucous membranes of the mouth, nose, four paws, and the inguinal incision turned from rosy to cyanotic; rectal temperature gradually decreased; arterial blood pressure initially increased compensatorily and then progressively declined; the ECG showed various arrhythmias such as tachycardia, bradycardia, and premature beats, eventually leading to ventricular fibrillation, mechanical dissociation, and electrocardiographic termination.

The criteria for cardiac arrest (CA) were: mean arterial pressure (MAP) ≤ 20 mmHg.

(3) Cardiopulmonary resuscitation: After 8 min of asphyxia, the tracheal tube was opened and the ventilator’s mechanical ventilation mode was activated with an FiO_2_ of 1.0. The tidal volume was set at 10 mL/kg, and the respiratory rate was adjusted to 80 breaths per minute. Chest compressions were initiated immediately. A single-hand three-finger method (thumb, index finger, and middle finger) was used for chest compressions, with the other hand’s thumb and index finger assisting in fixing the position of both axillae of the rat. The compression site was located at the midpoint of the line connecting both axillae on the rat’s thorax. The compression rate was set at 200 compressions per minute, with a depth of one-third the anteroposterior diameter of the thorax. Adrenaline was administered intravenously at a dose of 0.03 mg/kg, 30 s after the initiation of CPR, with additional intermittent doses of 0.01 to 0.03 mg/kg administered as needed. CPR was sustained until spontaneous circulation was restored, as evidenced by the emergence of normal QRS waves on the ECG and a mean arterial pressure (MAP) of at least 60 mmHg.

Criteria for successful CPR: Mean arterial pressure (MAP) must be ≥ 60 mmHg and sustained for over 10 min, accompanied by a gradual restoration of the rat’s lip and limb skin and mucous membranes to a pinkish hue.

Criteria for failed CPR: Absence of return of spontaneous circulation (ROSC) within 5 min or an inability to maintain MAP ≥ 60 mmHg for more than 10 min.

(4) Post-resuscitation management: Following successful resuscitation, a temperature-controlled blanket was employed to gradually elevate the rectal temperature to 35–37 °C. Mechanical ventilation with pure oxygen was sustained, and respiratory parameters were fine-tuned in accordance with the ETCO_2_ value to ensure it remained between 30–40 mmHg. Upon the rat resuming spontaneous breathing (60–120 breaths per minute) and exhibiting a pharyngeal reflex, the ventilator was disengaged. The rat was then observed for 5 min while breathing air. If vital signs remained stable, the tracheal tube was removed. Subsequently, the femoral artery and vein catheters were taken out and ligated, and the skin was disinfected using iodophor. A 10 mL/kg intraperitoneal injection of normal saline was administered to prevent dehydration. Once the rat regained consciousness and was able to move freely, it was returned to its cage, which was equipped with a 12-h light–dark cycle, and provided with readily available feed and water.

The criteria for failed weaning and extubation: After successful resuscitation and maintenance of mechanical ventilation for 1 h, the rat still could not be weaned and resume normal spontaneous breathing.

### 2.8 Intervention methods

The cardiopulmonary resuscitation model group received an intravenous injection of the same dose of heparin and normal saline immediately following CPR. The cardiopulmonary resuscitation heparin treatment group received an intravenous injection of 0.5 mg/kg of heparin immediately after CPR.

### 2.9 Observation indicators and methods

#### 2.9.1 Experimental timeline

**Figure 4:**
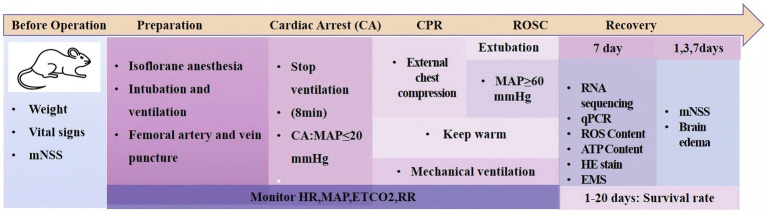
A comprehensive timeline of the experimental procedures from pre-operative preparation through recovery phase. The schematic outlines the sequential stages: baseline assessments (weight, vital signs, and mNSS), surgical preparation (anesthesia, intubation, and vascular access), cardiac arrest induction (8-min asphyxia), cardiopulmonary resuscitation, ROSC achievement, and the recovery period with specific time points for outcome assessments including mNSS at 1, 3, 7 days, brain edema measurement, and tissue collection for various analyses at 7 days post-ROSC, with survival monitoring extending to 20 days.

The comprehensive experimental timeline was designed to systematically evaluate both the acute and subacute phases of post-resuscitation injury and recovery. The study commenced with a pre-operative phase, during which baseline parameters, including body weight, vital signs, and mNSS scores, were recorded. This was immediately followed by the surgical and modeling phase on the same day, encompassing anesthesia, tracheal intubation, vascular access, the induction of cardiac arrest via 8-min asphyxia, and subsequent cardiopulmonary resuscitation. The post-ROSC phase involved longitudinal assessments: neurological function and cerebral edema were evaluated using mNSS scores and brain water content measurement, respectively, at Days 1, 3, and 7. On Day 7, a terminal time point, hippocampal tissues were collected from a subset of animals for comprehensive molecular and histological analyses, which included transcriptome sequencing, qPCR validation, HE staining, transmission electron microscopy, and the detection of ATP and ROS content. Concurrently, survival rates were monitored in a separate cohort of animals over a 20-day period to assess outcomes. The details were shown in Figure 4.

#### 2.9.2 Sample size for each experimental endpoint

The sample sizes for each specific experimental measurement were strategically determined to ensure robust and reliable data. Neurological function, assessed by mNSS scores at 1, 3, and 7 days post-ROSC, was evaluated in all surviving animals. This resulted in a sample size of n = 21 for the Sham group, n = 41 for the CPR group, and n = 41 for the CPR + HP group. Survival analysis was conducted on a dedicated subset, with n = 20 for both the CPR and CPR + HP groups, monitored for 20 days. The assessment of brain water content was performed at Days 1, 3, and 7. Finally, all comprehensive hippocampal tissue analyses—including RNA sequencing, qPCR, HE staining, electron microscopy, and ATP/ROS detection—were performed on tissues collected at the 7-day endpoint (Figures 1, 4).

#### 2.9.3 Modified neurological severity scores (mNSS) score

To evaluate the extent of neurological injury, the modified neurological severity scores (mNSS) scale was employed to assess the rats across the three groups, starting from the day of modeling and extending to the 7th day post-modeling. The mNSS scale comprises five components: a tail lift test (3 points), a straight walking test (3 points), a sensory test (2 points), a balance beam test (6 points), and a test for loss of reflex and abnormal movements (4 points), with a maximum total score of 18 points (refer to Table 1). Normal Sprague–Dawley (SD) rats score 0 points, scores ranging from 1 to 6 indicate mild neurological injury, 7 to 12 points signify moderate neurological injury, and 13 to 18 points denote severe neurological injury.

**Table 1:** Modified neurological deficit score criteria.

Test content	Score
**1. Motor tests**	
(1) Tail lift test	3
Forelimb flexion	1
Hindlimb flexion	1
Head movement >10° from the vertical axis (within 30s)	1
(2) Walking test (normal = 0; maximum = 3)	3
Normal walking	0
Unable to walk in a straight line	1
Circling towards the affected side	2
Falling towards the affected side	3
**2. Sensory tests**	2
(1) Tactile and visual test	1
(2) Proprioception test (Deep sensation, pushing the paw against the table edge to stimulate limb muscles)	1
(3) Beam balance test (normal = 0; maximum = 6)	6
Balances steadily	0
Grasps the side of the beam	1
Holds onto the beam, with one limb falling off	2
Holds onto the beam, with two limbs falling off or spins on the beam (>60 seconds)	3
Attempts to balance on the beam but falls (>40 seconds)	4
Attempts to balance on the beam but falls (>20 seconds)	5
Falls off without any attempt to grasp or maintain balance (<20 seconds)	6
**3. Absent reflexes and abnormal movements**	4
(1) Pinna reflex (head shaking when the ear canal is touched)	1
(2) Corneal reflex (lightly touching the cornea with cotton)	1
(3) Startle reflex (motor response triggered by noise)	1
(4) Seizures, myoclonus, dystonia	1
**Maximum total score**	18

#### 2.9.4 Brain tissue sampling

The skull was fixed, the cervical vertebrae were cut, and the left and right cranial bones were opened through the foramen magnum of the occipital bone to expose the intact brain. The cranial nerves were cut, and the whole brain was isolated. The tissue was then placed in a −80 °C freezer for cryopreservation until analysis.

#### 2.9.5 Hematoxylin-eosin (HE) staining

After fixation, rat brain tissue sections were prepared using either paraffin embedding or frozen sectioning. The paraffin sections underwent decalcification in environmentally friendly solutions I and II for 20 min each, absolute ethanol I and II for 5 min each, 75% alcohol for 5 min, and were then rinsed with water. Frozen sections were removed from −20 °C storage, rewarmed, fixed in tissue fixative for 15 min, and subsequently rinsed with water. All sections were then treated with a high-definition constant staining pretreatment solution for 1 min, stained with hematoxylin for 3–5 min, rinsed with water, differentiated with a differentiation solution, rinsed again, re-blueed with a re-blueing solution, and finally rinsed with water. The dehydration and transparency process involved treatment with 95% alcohol I, II, III for 2 min each, n-butanol I, II for 2 min each, and xylene I, II for 2 min each. Following dehydration and transparency, the sections were mounted with neutral gum, examined under a microscope, and subjected to image acquisition analysis.

#### 2.9.6 Water content of brain tissue

Approximately 200 mg of frozen brain tissue from the parietal lobe was obtained and allowed to thaw at room temperature. The wet weight was determined using a precision electronic analytical balance (AR224CN, with an accuracy of 0.0001 g). Subsequently, the tissue was placed in an electric constant temperature drying oven (GRX-20A) set at 110 °C for 12 h to dry. The dry weight was then measured, and the water content of the brain tissue was calculated using the dry-to-wet weight ratio method: water content of brain tissue = (wet weight − dry weight)/wet weight × 100%.

#### 2.9.7 qRT-PCR

(1) Extraction of Total RNA from Tissue.

Approximately 15–30 mg of brain tissue was taken and homogenized with TRK Lysis Buffer using a homogenizer. The homogenate was transferred to a homogenization column and centrifuged at 14,000×*g* at room temperature for 2 min. The filtrate was collected. An equal volume of 70% ethanol was added, mixed well, and transferred to a binding column placed in a collection tube. Centrifugation was performed at 10,000 × *g* at room temperature for 1 min, and the filtrate was discarded. Then, 500 μL of RNA Wash Buffer I was added to the binding column, which was placed in the collection tube, and centrifuged at 10,000 × *g* for 1 min. The filtrate was discarded. Next, 500 μL of RNA Wash Buffer II was added to the binding column and centrifuged at 10,000 × *g* for 1 min. The filtrate was discarded. RNA Wash Buffer II was used for two washes. The RNA binding column was then placed in a collection tube and centrifuged at 10,000 × *g* for 2 min. Subsequently, 50 μL of Nuclease-free Water was added to the binding column, with the volume of Nuclease-free Water adjusted according to the RNA concentration. Finally, centrifugation was performed at 10,000 × *g* for 2 min to elute the RNA into a new centrifuge tube. The extracted RNA was quantified using the NANODROP (Thermo Fisher) to quickly determine the RNA concentration of the sample. The absorbance ratios at 260/280 and 260/230 were also measured. Following calibration, the sample was stored at −80 °C in a refrigerator.

(2) Primer design.

We searched for the corresponding species’ gene ID on NCBI and then input the relevant gene into Primer-BLAST to design primers. The primers were designed to be 17–25 bp in length, with a GC content of 45–55%, and to produce a quantitative product of 80–220 bp. The corresponding primer sequences, as displayed in the table below, were synthesized by Sangon Biotech.

**Table:** 

*Epsa1*-F	GAGGTAGATGAGCAGCGTGAC
*Epsa1*-R	AGAGCATACTGGAGCGGAAGGAG
*Idh2*-F	TCCTGTTTGCTGATGCCCTTTTCC
*Idh2*-R	CTCACCAAGCCCAAGCCTCAAG
*ACTB*-F	CTGTTGCTGTAGCCATATTCATTG
*ACTB*-R	AGGTTGTCTCCTGTGACTTCAA
*Hif3a*-F	GCCTCAAGCTGCCTCACCTTAT
*Hif3a*-R	CACCGACTCGCTAGACACCCTA

(3) Reverse transcription and qPCR amplification.

We diluted the primers to a concentration of 500–1,000 μM using low-EDTA TE Buffer, based on their measured OD values. We then assembled a 20 μL reverse-transcription reaction in an eight-tube strip (with caps), utilizing the TaKaRa reverse-transcription kit, and loaded 300–500 ng of total RNA into each tube. The reverse-transcription program we executed consisted of incubation at 37 °C for 15 min, followed by 85 °C for 5 s, and concluded with storage at 4 °C. Subsequently, we prepared 20 μL qPCR reactions using the TaKaRa amplification kit, each containing Taq SYBR Green qPCR Master Mix, Primer-F, Primer-R, the corresponding reverse-transcription product, and ddH₂O. We conducted amplification on a QuantStudio 5 instrument using a two-step protocol: an initial cycle of pre-denaturation at 95 °C for 30 s, followed by 40 cycles of 95 °C for 10 s and 60 °C for 30 s (with fluorescence acquisition). Finally, we obtained the melting curve using the instrument’s default program.

#### 2.9.8 Transmission electron microscopy

Fresh tissue was cut into 1 mm^3^ cubes within 1–3 min and immediately immersed in pre-cooled electron microscopy fixative. The cubes were then trimmed to standard size with a scalpel in the same solution and transferred to an EP tube containing fresh fixative, and stored at 4 °C. The samples were washed three times for 15 min each in 0.1 M PB (pH 7.4), followed by fixation in 1% osmium acid (prepared in PB) at room temperature in the dark for 2 h. They were then washed three times for 15 min each under the same conditions. The samples were dehydrated in a gradient of 30–50%-70–80%-95–100-100% ethanol for 20 min each at room temperature, followed by two 15-min washes in 100% acetone. The samples were embedded successively in acetone:812 embedding agent at a ratio of 1:1 for 2–4 h at 37 °C, 1:2 overnight at 37 °C, and pure 812 for 5–8 h at 37 °C. The samples were placed in embedding molds and covered with pure 812, and then kept in a 37 °C oven overnight and polymerized at 60 °C for 48 h to obtain resin blocks. Semi-thin sections of 1.5 μm were cut with a semi-thin sectioning machine and stained with toluidine blue for light microscopy localization. Ultra-thin sections of 60–80 nm were cut with an ultrathin sectioning machine and collected on 150-mesh copper grids. The copper grids were successively treated with 2% uranyl acetate in alcohol for 8 min in the dark, 70% ethanol three times, pure water three times, 2.6% lead citrate for 8 min in the absence of CO₂, and pure water three times. After blotting dry with filter paper, they were air-dried at room temperature overnight. The samples were finally observed and imaged for analysis under a transmission electron microscope.

#### 2.9.9 ATP content detection

(1) Sample preparation:

For tissue samples, 0.1 g of tissue was homogenized with 0.9 mL of lysis buffer (1:9, weight/volume) for 5–10 min, then heated in boiling water for 2 min, cooled under running water, and centrifuged at 4 °C at 10,000 × *g* for 10–15 min. The supernatant was collected and placed on ice. For cell samples, approximately 2 × 10^6^ cells were resuspended in 200–300 μL of lysis buffer, homogenized for 5 min, heated in boiling water for 2 min, cooled, and centrifuged to collect the supernatant.

(2) Detection preparation:

The 200 μM ATP standard solution was diluted in ultrapure water to yield concentrations of 4, 2, 1, 0.5, 0.25, and 0.125 μM. The ATP detection working solution was prepared by combining the ATP detection enzyme reagent with the detection buffer at a ratio of 1:50 (v/v) on-site.

(3) Detection reaction:

100 μL of the ATP detection working solution was added to the standard, blank, and sample tubes. The tubes were then permitted to stand at room temperature for 5 min. Subsequently, 20 μL of the ATP standard solution was introduced into the standard tube, 20 μL of ultrapure water was added to the blank tube, and 20 μL of the supernatant to be tested was dispensed into the sample tube. The plate was agitated for 15 s or thoroughly mixed, and the value was measured using a chemiluminescence microplate reader.

(4) Data processing:

Excel was utilized to plot the standard curve, with the difference between the standard group value and the blank group value as Y and the ATP concentration (μmol/L) as X. The ATP content in tissues (μmol/kg) was calculated using the formula: [(sample group value − blank group value) − b]/a × V/m × f. Here, V represents the volume of the lysis buffer (L), m is the wet weight of the tissue (kg), and f is the dilution factor. The ATP content in cells (μmol/10^6^ cells) was calculated using the formula: [(sample group value − blank group value) − b]/a × V/n × f, where n is the number of cells (10^6^).

#### 2.9.10 Detection of reactive oxygen species (ROS) in tissues

(1) Preparation of 100 × DHE staining solution: Mix 2 μL of DHE probe (500×) thoroughly with 8 μL of PBS buffer to prepare the 100 × DHE staining solution, which should be kept on ice until use.
(2) Sample processing: Fresh tissue samples were collected and washed with PBS. To 50 mg of tissue, add 450 μL of homogenization buffer and homogenize the mixture thoroughly. Centrifuge the homogenate at 10,000*g* for 10 min at 4 °C, discard the pellet, and use the supernatant immediately.
(3) Sample Detection: To each well of a black 96-well plate, 90 μL of homogenization buffer was added, followed by 10 μL of tissue supernatant. Subsequently, 1 μL of the 100 × DHE staining solution was introduced. The plate was then incubated at 37 °C in the dark for 20 min. Fluorescence was measured using a microplate reader, with excitation at 520 nm and emission at 605 nm.
(4) Data Analysis: ROS levels in the tissue were expressed as fluorescence intensity (RFU) per unit of protein mass (protein concentration multiplied by sample volume).

### 2.10 Transcriptome sequencing and data analysis

RNA sequence analysis was conducted on the collected samples. Total RNA was extracted using an animal RNA extraction kit following the manufacturer’s instructions. The concentration of the extracted nucleic acids was measured using a Nanodrop 2000 (Thermo Fisher), and the integrity of the RNA was assessed using an Agilent 2100 Bioanalyzer and LabChip GX (PerkinElmer). The qualified total RNA was then further purified using a nucleic acid purification kit. The sequencing library was constructed and sequenced using an Illumina Novaseq6000 by BGI. Inter-group differential expression analysis was performed using R software to analyze the gene expression profiles of samples in each group. Following correction through statistical tests, genes with a *p*-value less than 0.05 were identified as differentially expressed genes (DEGs).

#### 2.10.1 Establishment of mitochondrial gene database

By using “mitochondrion” and “rattus norvegicus” as keywords and searching the NCBI database,^1^ a rat mitochondrial gene dataset was established.

#### 2.10.2 Mapping of disease targets and differentially expressed genes

The intersection between the differentially expressed genes (DEGs) and the rat mitochondrial gene dataset was analyzed to identify potential targets of heparin-regulated mitochondrial genes, and a Venn diagram was subsequently created.

#### 2.10.3 GO and KEGG enrichment analysis of differentially expressed genes

The potential targets underwent KEGG and GO enrichment analysis via the XianTao online database. In this study, *P <* 0.05 was utilized as the significance threshold. The Cellular Component (CC), Biological Process (BP), and Molecular Function (MF) categories under the GO annotation were chosen to characterize the CC, BP, and MF of the differentially expressed genes (DEGs). The biological functions and pathways with greater relevance were filtered and bar charts were generated to illustrate these findings.

#### 2.10.4 Protein interaction network analysis of key target proteins

The STRING online analysis platform was used to construct a protein interaction network relationship diagram of the potential targets related to heparin-regulated mitochondria.

### 2.11 Statistical methods

Data analysis and statistics were performed using GraphPad Prism 9.5 software. The p < 0.05 was considered statistically significant. The data of qPCR, cerebral water content changes post-ROSC of CA-CPR rats, and ATP and ROS contents in hippocampal tissues of rats were analyzed by ordinary one-way ANOVA with multiple comparisons. The data of mNSS score were analyzed by ordinary two-way ANOVA with multiple comparisons. The data of 20-day survival rates were analyzed by Gehan-Breslow-Wilcoxon test.

## 3 Results

### 3.1 Animal model overview, experimental timeline, and sample sizes

A total of 112 male Sprague–Dawley rats were included in this study. The experimental design and animal allocation are summarized in Figure 1. Following model establishment, the rates of return of spontaneous circulation (ROSC) were 89.1% (41/46) in the CPR group and 91.1% (41/45) in the CPR + Heparin (CPR + HP) group, indicating comparable model success rates between groups.

The detailed procedural steps for establishing the asphyxial cardiac arrest model, including anesthesia, monitoring, cardiac arrest induction, resuscitation, and post-ROSC management, are illustrated in Figures 2, 3. The comprehensive experimental timeline, outlining all assessment time points from pre-operative preparation through the recovery phase, is presented in Figure 4.

Functional and survival assessments were conducted as follows: Neurological function was evaluated using modified Neurological Severity Scores (mNSS) at 1, 3, and 7 days post-ROSC in all surviving animals (Sham: n = 21; CPR: n = 41; CPR + HP: n = 41). The 20-day survival rate was monitored in a dedicated subset of animals (CPR: n = 20; CPR + HP: n = 20).

Tissue collection and analysis were performed at specified time points. Cerebral edema was assessed by measuring brain water content at 1, 3, and 7 days post-ROSC. For comprehensive molecular and histological analyses, hippocampal tissues were collected at 7 days post-ROSC from a subset of animals that had completed the mNSS assessments. These analyses included transcriptome sequencing, qPCR validation, HE staining, transmission electron microscopy, ATP content detection, and ROS content detection.

### 3.2 Differential expression genes (DEGs) screening results in three rat groups

**Figure 5:**
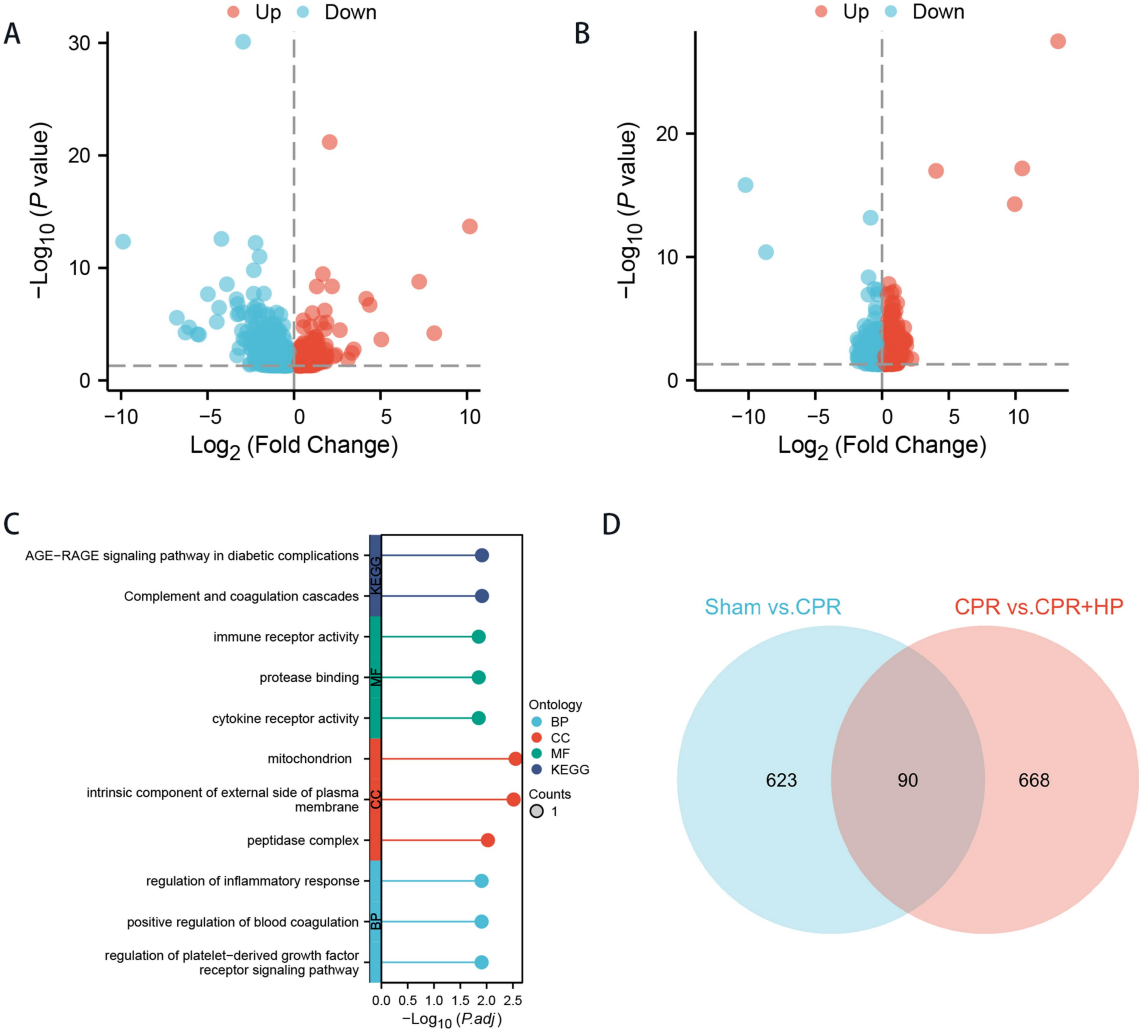
The identification of differentially expressed genes (DEGs) and functional enrichment analysis among the cardiac arrest-cardiopulmonary resuscitation model group, heparin treatment group, and sham group. **(A)** The volcano plot displays 711 DEGs between the cardiac arrest-cardiopulmonary resuscitation model group and the sham group. **(B)** The volcano plot presents 757 DEGs between the cardiac arrest-cardiopulmonary resuscitation model group and the heparin treatment group. **(C)** Functional enrichment analysis reveals significant associations between DEGs and mitochondrial pathways. The enrichment analysis of DEGs was conducted using KEGG **(B)** and GO **(C)** methodologies. **(D)** The Venn diagram demonstrates 90 co-expressed genes obtained from the intersection of DEGs between the two comparative groups.

The Model Group (CPR Group), the Heparin-Treated Group (CPR + HP Group), and the Sham Group. Compared to the Sham Group, 711 differentially expressed genes (DEGs) were identified in the hippocampal tissues of the Model Group, consisting of 255 upregulated and 456 downregulated genes. In contrast, 757 DEGs were detected in the Heparin-Treated Group compared to the Model Group, including 255 upregulated and 502 downregulated genes, as detailed in Figures 5A,B.

Venn diagram analysis revealed 90 co-expressed genes among the Sham, Model, and Heparin-Treated Groups. The intersection of DEGs between the Sham/Model Groups and the Model/Heparin-Treated Groups was further visualized via a Venn diagram generated using the Xiantao Online Database, yielding 90 differentially expressed genes (Figure 5D).

KEGG and GO analyses were conducted on differentially expressed genes (DEGs) among the model group, treatment group, and Sham Group using Xiantao academic data (Figure 5C). The KEGG pathway analysis indicated significant enrichment in the AGE-RAGE signaling pathway and complement/coagulation cascades (Figure 5C). The GO enrichment results showed that the therapeutic effects of heparin on cardiac arrest, following cardiopulmonary resuscitation, primarily involved mitochondrial-related genes, with biological processes significantly associated with inflammatory responses and growth factor signaling pathways.

**Figure 6:**
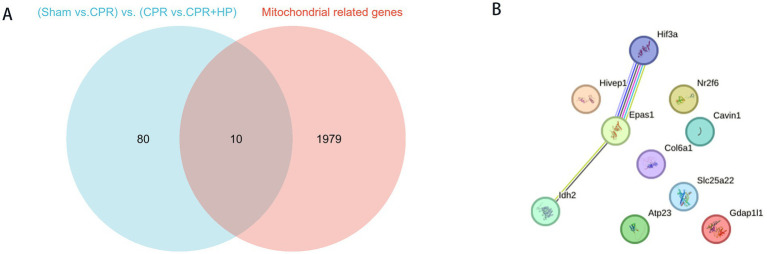
Venn diagram and PPI network interaction map. **(A)** The intersection analysis between co-expressed genes across three groups and the mitochondrial gene set identified 10 mitochondria-related genes. **(B)** Protein–protein interaction network analysis revealed three pivotal genes: *Epsa1*, *Idh2*, and *Hif3a*.

A comprehensive search of the NCBI database identified 1,989 genes associated with the regulation of mitochondrial function. Subsequent mapping of these mitochondrial-related genes with differentially expressed genes (DEGs) from sequencing data yielded 10 overlapping genes, which are visualized in a Venn diagram (Figure 6A).

Protein–protein interaction (PPI) network construction and hub gene analysis were conducted using the STRING database with medium confidence (interaction score ≥ 0.40). This analysis identified three key genes—*Epsa1*, *Idh2*, and *Hif3a*—with *Epsa1* exhibiting the highest centrality (Figure 6B).

### 3.3 ROC analysis

**Figure 7:**
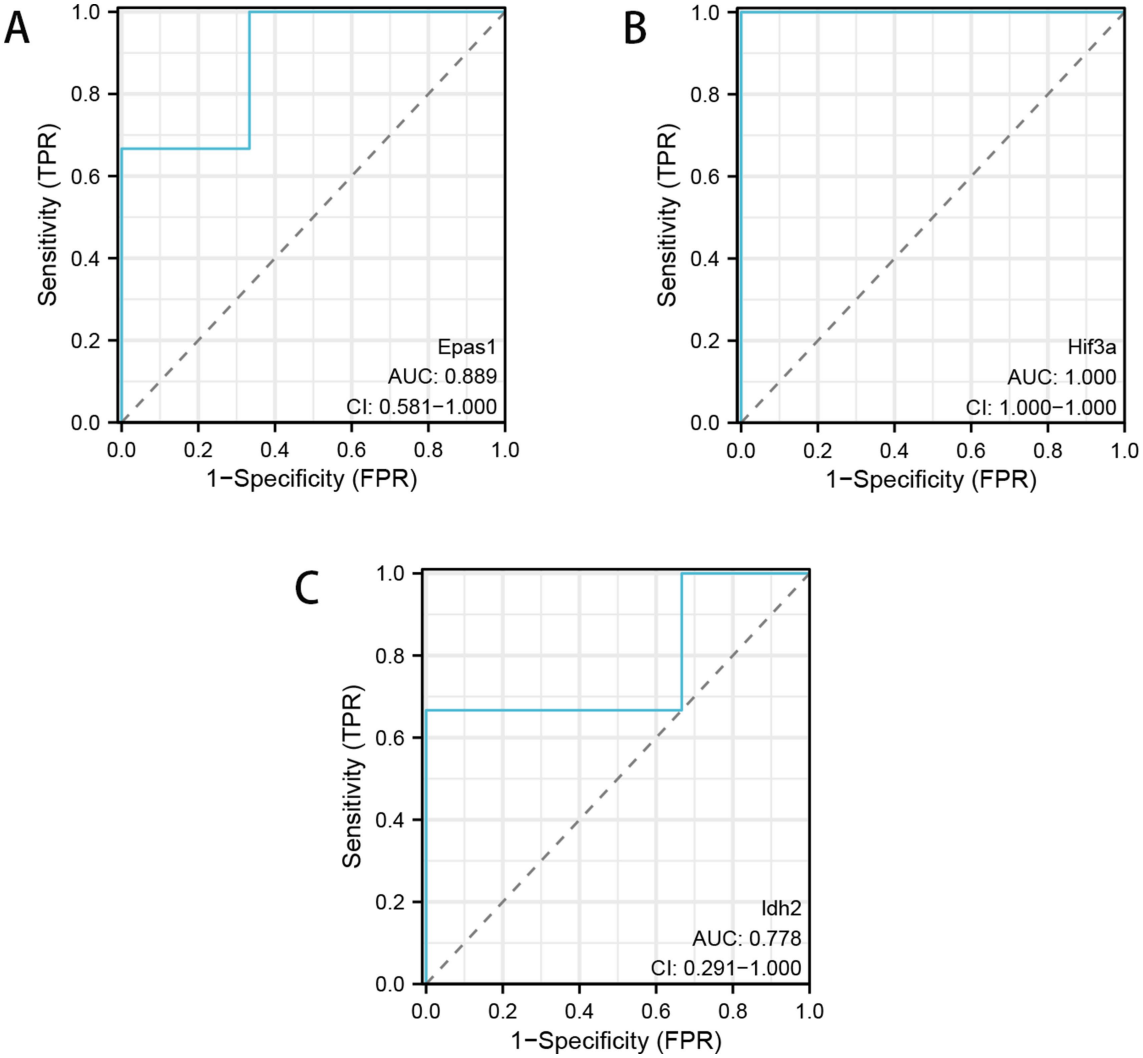
ROC curves of *Epsa1*, *Idh2* and *Hif3a* for diagnosing the differences between the cardiac arrest cardiopulmonary resuscitation model group and the sham group. **(A)** ROC analysis of *Epsa1*. **(B)** ROC analysis of *Hif3a*. **(C)** ROC analysis of *Idh2*.

To determine the potential value of the three key genes, *Epsa1*, *Idh2*, and *Hif3a*, in regulating the mitochondria of hippocampal neurons in rats after heparin-induced cardiac arrest and cardiopulmonary resuscitation, we conducted a receiver operating characteristic (ROC) analysis. In the sequencing results, the area under the curve (AUC) for *Epsa1* was greater than 0.8, for *Idh2* was greater than 0.75, and for *Hif3a* was greater than 0.9 (Figure 7). Overall, *Epsa1*, *Hif3a*, and *Idh2* demonstrated significant diagnostic value, suggesting that they could be considered as potential drug targets for diagnosing the regulation of hippocampal neuron mitochondria in rats after heparin-induced cardiac arrest and cardiopulmonary resuscitation.

### 3.4 qPCR validation

**Figure 8:**
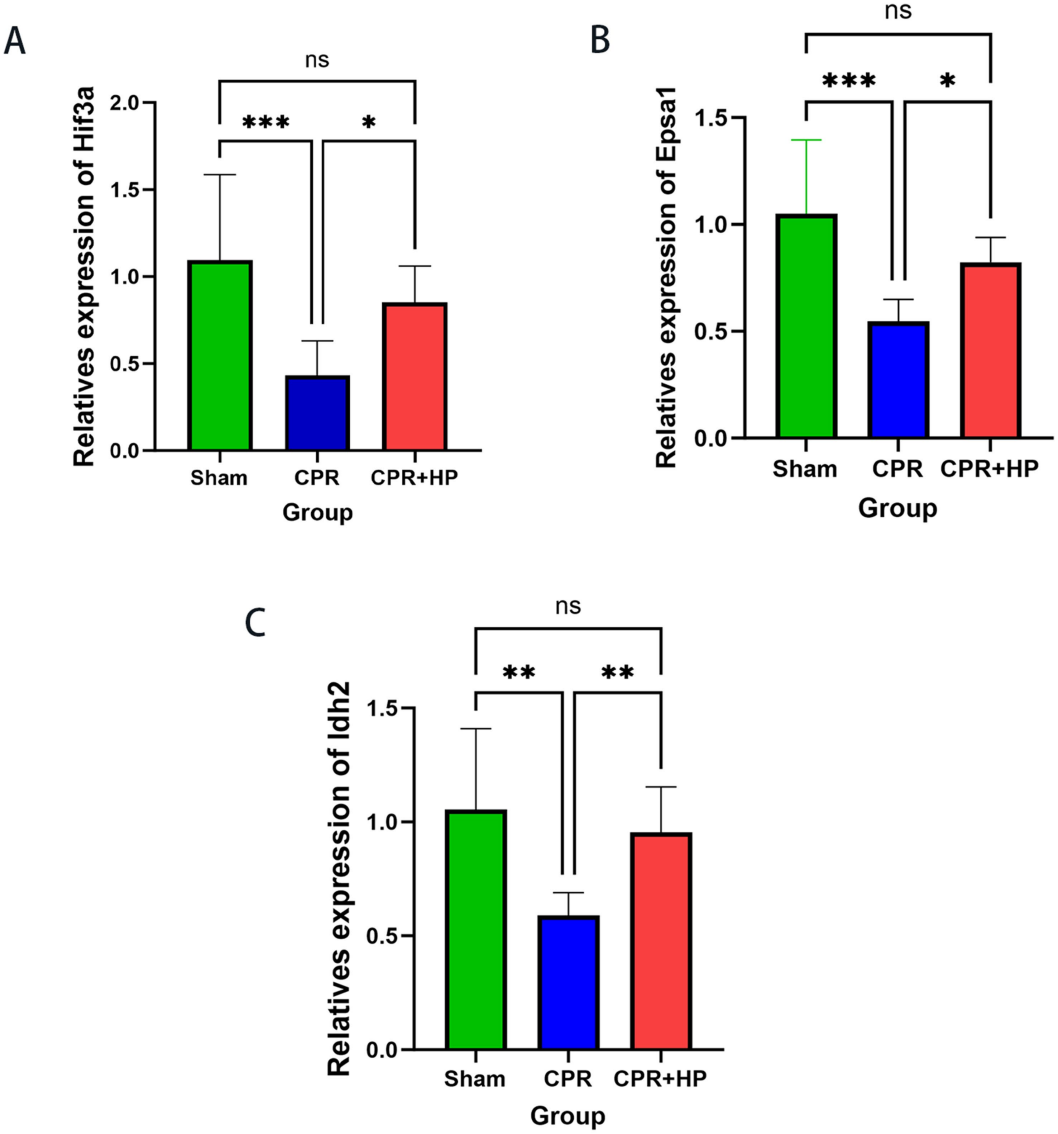
Effects of heparin on mRNA expression levels of *Epsa1*, *Idh2*, and *Hif3a* post-modeling. **(A)** Heparin’s impact on *Epsa1* mRNA expression following cardiopulmonary resuscitation, **(B)** *Idh2* mRNA expression, and **(C)** *Hif3a* mRNA expression. ns *p* > 0.05, ***p < 0.001, ****p < 0.0001. Compared to the sham group, the model group exhibited significantly reduced relative mRNA expression levels of *Epsa1*, *Idh2*, and *Hif3a* (*p <* 0.05). In contrast to the model group, the heparin treatment group demonstrated significantly elevated relative mRNA expression levels of *Epsa1*, *Idh2*, and *Hif3a* (*p <* 0.05). The data were analyzed using the one-way ANOVA with multiple comparisons.

Quantitative PCR analysis revealed significantly reduced relative mRNA expression levels of *Epsa1*, *Idh2*, and *Hif3a* in the hippocampal tissues of the model group compared to the sham group (*p <* 0.05). In contrast, the heparin-treated group exhibited a marked upregulation in the relative mRNA expression levels of these genes (*Epsa1*, *Idh2*, and *Hif3a*) compared to the model group (*p <* 0.05). Detailed results are presented in Figure 8.

**Figure 9:**
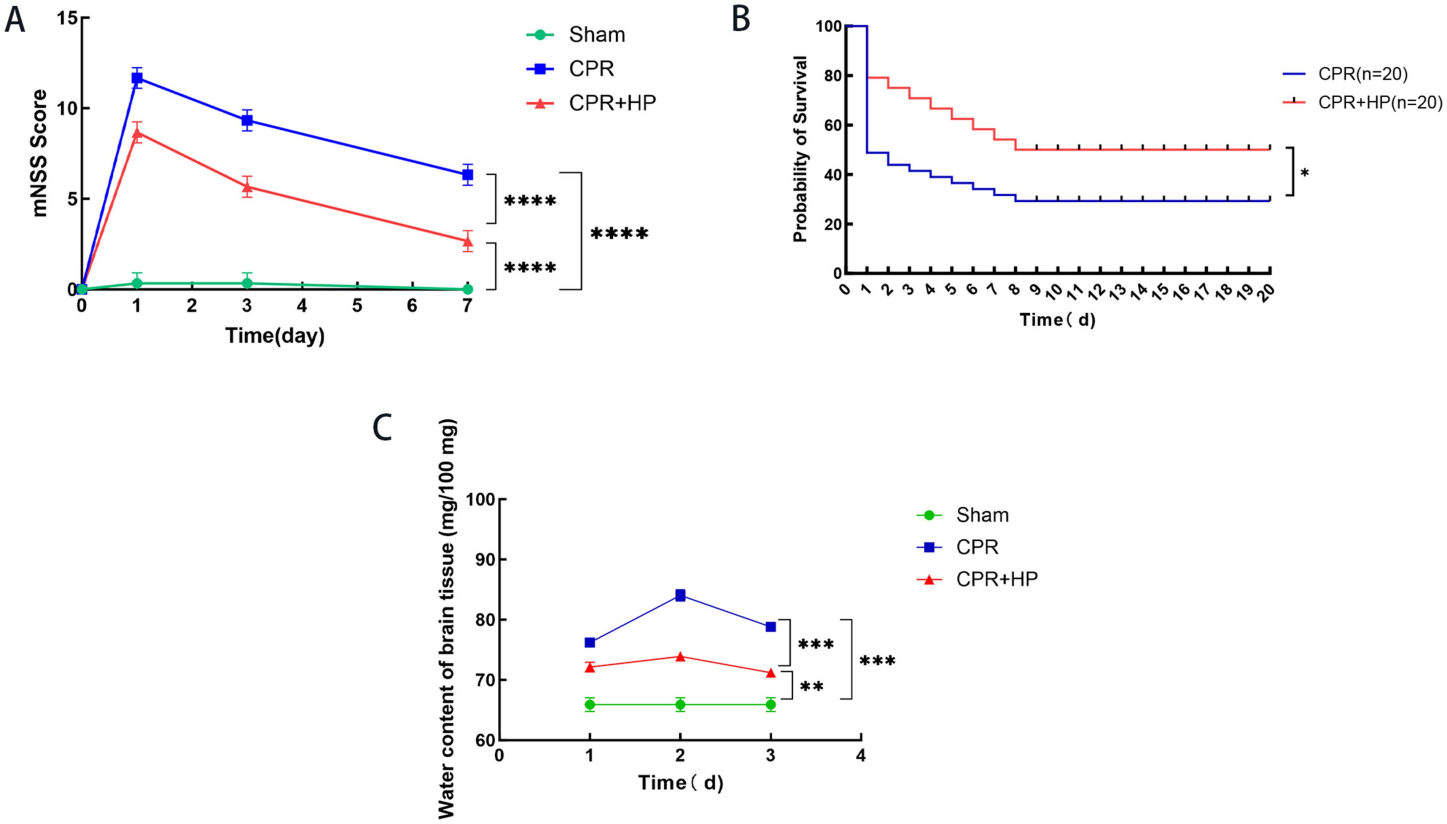
mNSS scores, 20-day survival rates and cerebral water content changes post-ROSC of CA-CPR rats. **(A)** The effect of heparin on mNSS scores in CA-CPR rats. Compared with the CPR group, the CPR + HP group exhibited a significant reduction in 7-day mNSS scores (*p <* 0.05). Relative to the sham group, the CPR group showed markedly elevated 7-day mNSS scores (*p <* 0.05), while the CPR + HP group also demonstrated increased scores compared to the Sham Group (*p* < 0.05). **(B)** Kaplan–Meier analysis of 20-day survival rates post-ROSC in the two groups (*p* < 0.05). **(C)** Trend analysis of cerebral water content changes across the three experimental groups at baseline (pre-resuscitation), and on post-resuscitation days 1, 3, and 7. The data of mNSS score were analyzed by ordinary two-way ANOVA with multiple comparisons. The data of cerebral water content changes post-ROSC of CA-CPR rats were analyzed by ordinary one-way ANOVA with multiple comparisons. The data of 20-day survival rates were analyzed by Gehan-Breslow-Wilcoxon test.

Neurological function impairment was assessed using the modified Neurological Severity Score (mNSS) at 1, 3, and 7 days post-return of spontaneous circulation (ROSC) in the three experimental groups (Figure 9A). All rats exhibited baseline mNSS scores of 0 prior to hypoxia. Rats in the Sham Group showed minor alterations in balance beam performance on the mNSS scale 1 day after anesthesia and invasive procedures. The CPR and CPR + HP groups demonstrated the most severe neurological impairment on day 1 post-ROSC, with a gradual recovery to mild impairment by days 3–7. Compared to the CPR group, heparin-treated rats exhibited significantly lower mNSS scores at 1, 3, and 7 days post-ROSC (*p < 0.05*), as detailed in Figure 9A. These findings indicate that heparin administration ameliorates neurological injury following CA-CPR-induced ROSC.

As illustrated in Figure 9B, the 20-day survival rates post-ROSC were 30% (6/20) in the CPR group and 55% (11/20) in the CPR + HP group, demonstrating that heparin administration significantly improved the 20-day survival rate in CPR-treated rats (p < 0.05).

As presented in Table 2, the cerebral water content across groups at various time points was as follows: The Sham Group exhibited a baseline value of 64.73 ± 4.03 mg/100 mg. In the CPR (Model) Group, cerebral water content measured 75.6 ± 1.80 mg/100 mg, 83.12 ± 2.96 mg/100 mg, and 78.46 ± 1.87 mg/100 mg at 1, 3, and 7 days post-resuscitation, respectively. Conversely, the CPR + HP (Heparin) Group showed values of 71.42 ± 2.11 mg/100 mg, 73.23 ± 1.32 mg/100 mg, and 70.64 ± 1.86 mg/100 mg at 1, 2, and 3 days post-resuscitation.

**Table 2:** Brain tissue water content in different groups after resuscitation (mg/100 mg).

Group Time	Sham	CA-CPR	CA-CPR-Heparin
Day1	64.73±4.03	75.6±1.80	71.42±2.11
Day3	-	83.12±2.96	73.23±1.32
Day7	-	78.46±1.87	70.64±1.86

One day after resuscitation, the comparisons of brain tissue water content among the three groups (Sham vs. CPR; Sham vs. CPR + HP; CPR vs. CPR + HP) all showed statistically significant differences (*p* < 0.05). Three days after resuscitation, the comparisons of brain tissue water content among the three groups (Sham vs. CPR; Sham vs. CPR + HP; CPR vs. CPR + HP) all showed statistically significant differences (*p* < 0.05). Seven days after resuscitation, the comparisons of brain tissue water content among the three groups (Sham vs. CPR; Sham vs. CPR + HP; CPR vs. CPR + HP) all showed statistically significant differences (*p* < 0.05). Shown in Table 2 and Figure 9C. The data were analyzed using the one-way ANOVA with multiple comparisons.

### 3.5 Results of HE staining

**Figure 10:**
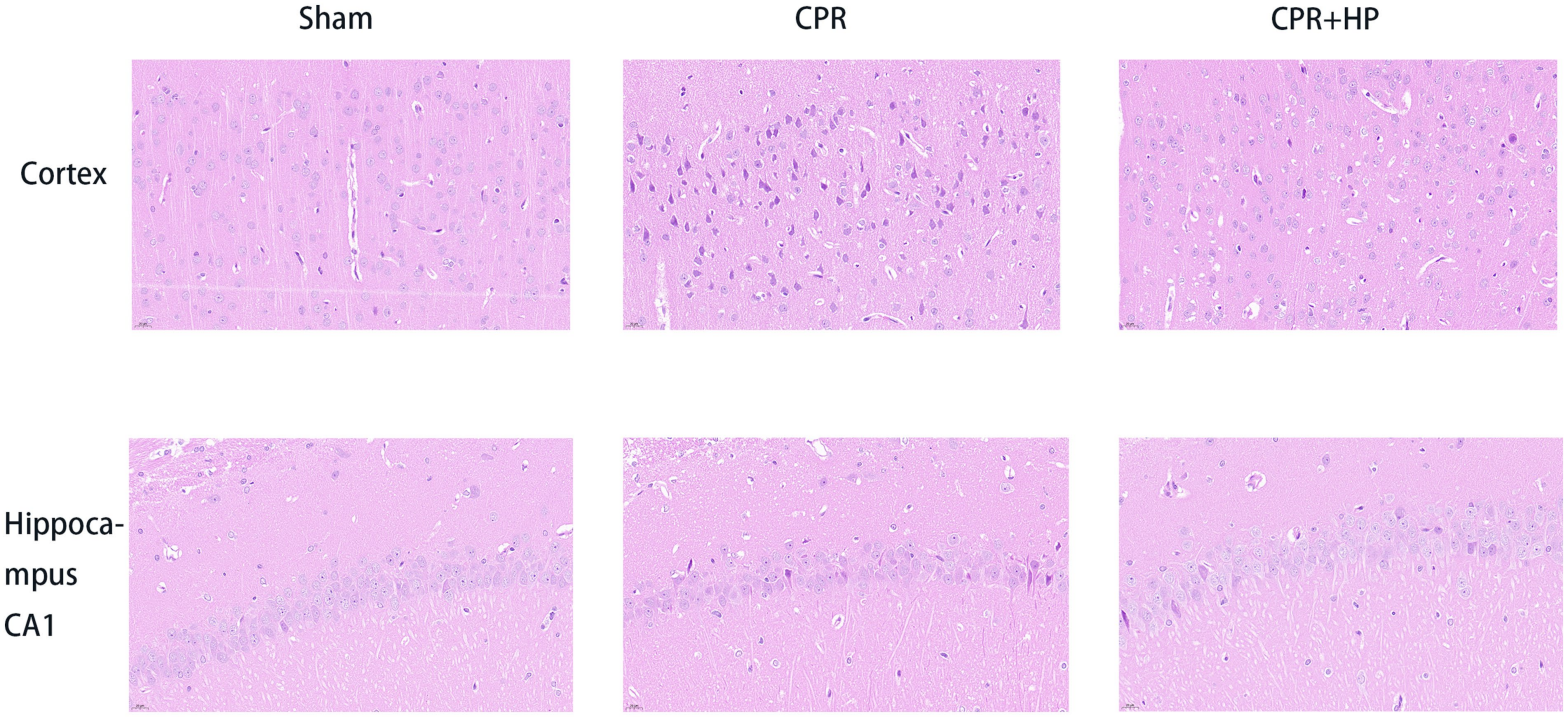
Protective effects of heparin on neuronal morphology in the cerebral cortex and hippocampal CA1 region of CPR rats (HE staining, scale bar = 20 μm). Sham group: Neurons exhibited orderly arrangement with large, rounded nuclei, uniform staining, and distinct Nissl bodies. CPR group: Reduced neuronal density was observed, accompanied by shrunken somata, pyknotic and hyperchromatic nuclei, widened intercellular spaces, and evident vacuolar degeneration. CPR + HP group: Neuronal damage was significantly mitigated, showing relatively organized cellular arrangement, near-normal nuclear morphology, and reduced vacuolar degeneration.

HE staining results of the cerebral cortex and hippocampal CA1 region in the three groups of rats are shown in Figure 10. Compared with the Sham Group, varying degrees of neuronal damage were observed in the cerebral cortex and hippocampal CA1 region of the model group (CPR) and the heparin-treated group (CPR + HP) after resuscitation. In the CPR group, neurons exhibited disorganized arrangement, irregular cell sizes, partially visible pyknotic nuclei, and initial glial cell proliferation. Immediate heparin administration postresuscitation mitigated neuronal damage following the restoration of spontaneous circulation.The pathological changes characterized by disorganized neuronal arrangement, heterogeneous cell sizes, partial pyknotic nuclei, and incipient glial hyperplasia were significantly ameliorated by immediate heparin administration.

### 3.6 Results of electron microscopic observation of mitochondria in hippocampal tissue

**Figure 11:**
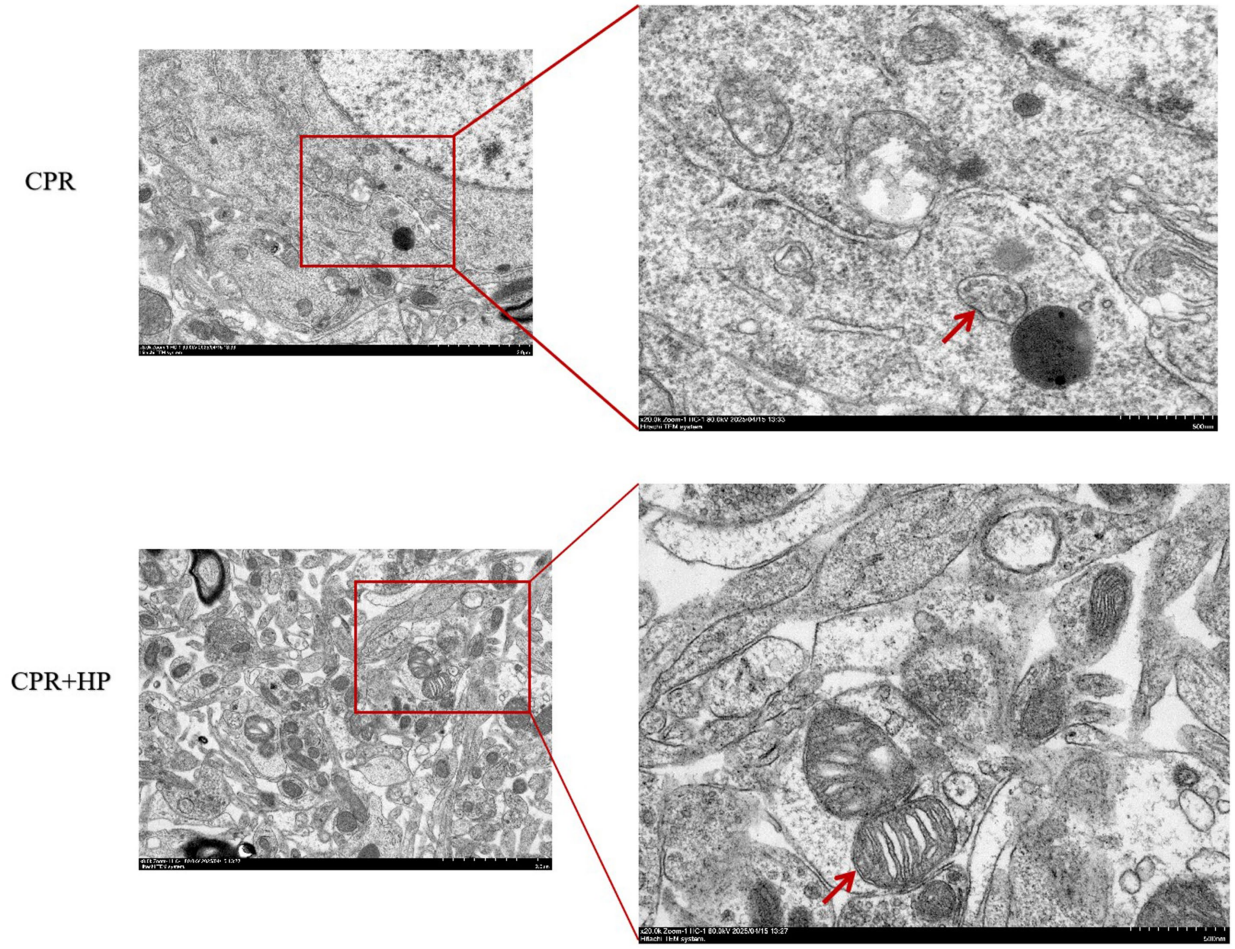
Effects of heparin on the ultrastructure of neurons under electron microscopy. Red arrows indicate mitochondria. Magnification: 8,000×, white scale bar = 2 μm; 20,000×, white scale bar = 500 nm.

The ultrastructure of hippocampal neurons was observed under a transmission electron microscope, and the results are shown in Figure 11. In the model group (CPR), the neurons were shrunken, with irregular morphology, the nuclei were shriveled, the nuclear membranes were uneven and unclear, the heterochromatin was aggregated in the nucleus, the mitochondria were atrophied, the mitochondrial cristae were reduced or even disappeared, and some mitochondria showed rupture of the outer membrane. In the heparin treatment group (CPR + HP), the neurons had a more regular morphology compared to the CPR group, the nuclear membranes were relatively smooth, the volume of mitochondria was restored, and the density of mitochondrial cristae increased.

### 3.7 Biochemical detection of ATP content in rat hippocampal tissue

**Figure 12:**
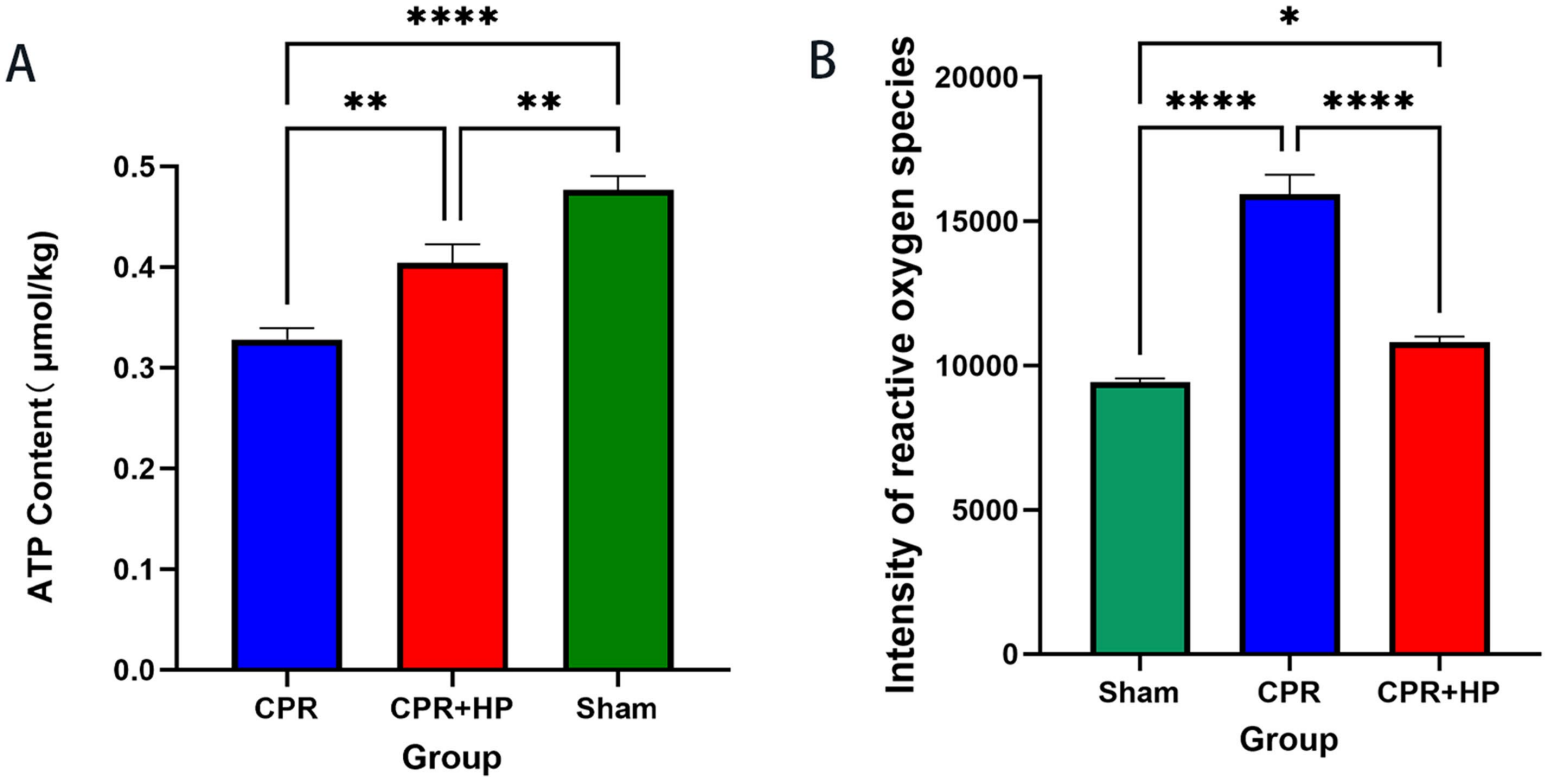
ATP content and ROS content in hippocampal tissues of rats in each groups. **(A)** ATP content in hippocampal tissues of rats across experimental groups (biochemical assay). Compared with the sham Group, the CPR group exhibited significantly reduced ATP levels (*p < 0.05). Relative to the CPR group, the CPR + HP group demonstrated a marked increase in ATP content (**p < 0.05). **(B)** ROS content in hippocampal tissues of rats across experimental groups (biochemical assay). The CPR group showed significantly elevated ROS levels versus the sham Group (*p < 0.05), while the CPR + HP group displayed substantial reduction in ROS content compared to the CPR group (**p < 0.05). The data were analyzed using the one-way ANOVA with multiple comparisons.

Biochemical detection was conducted on the ATP content in the hippocampal tissues of the three groups of rats. Compared with the sham group, the ATP level in the model group was significantly decreased (*p* < 0.05). Compared with the model group, the ATP content in the treatment group was significantly increased (*p* < 0.05), as shown in Figure 12A.

### 3.8 Biochemical detection of ROS content in rat hippocampal tissue

Biochemical detection was carried out on the ROS content in the hippocampal tissues of the three groups of rats. Compared with the sham group, the ROS level in the model group was significantly increased (*p <* 0.05). Compared with the model group, the ROS content in the treatment group was significantly decreased (*p <* 0.05), as shown in Figure 12B.

## 4 Discussion

Heparin, a classical anticoagulant with well-established clinical safety and efficacy for the prevention and treatment of thrombotic diseases (23, 24), has not been systematically investigated for its neuroprotective properties during cardiopulmonary resuscitation (CPR) following cardiac arrest (CA). Utilizing an asphyxia-induced CA-CPR rat model, we demonstrated that the immediate administration of a low-dose of heparin (0.5 mg/kg) upon restoration of spontaneous circulation (ROSC) significantly improved neurological function, reduced cerebral edema, and enhanced long-term survival, providing significant experimental evidence supporting its potential clinical application. More importantly, through integrated RNA sequencing and mechanistic investigations, we uncovered that the essence of heparin’s neuroprotective effect is its multi-dimensional regulation of mitochondrial dysfunction.

Transcriptomic analysis revealed that genes regulated by heparin were significantly enriched in mitochondria-related pathways. Protein–protein interaction network analysis further pinpointed this effect on three hub genes: *Epsa1*, *Idh2*, and *Hif3a*. These three genes correspond to three core dimensions of heparin’s protective effects: mitochondrial structural integrity, antioxidant defense capacity, and metabolic homeostasis regulation. Their synergistic action collectively reverses ischemia–reperfusion-induced mitochondrial damage, constituting the molecular foundation of heparin’s neuroprotection.

At the mechanistic level, the coordinated upregulation of these three genes systemically improved mitochondrial function across three dimensions: mitochondrial structure, energy metabolism, and metabolic reprogramming. *Epsa1*, as a key regulator of mitochondrial network stability, prevented the aberrant opening of the mitochondrial permeability transition pore (mPTP) by inhibiting *Drp1*-mediated excessive fission. This action maintained mitochondrial membrane potential, driving ATP synthesis while reducing electron leakage from the electron transport chain, thereby curbing ROS generation at its source (25–27).

*Idh2*, central to mitochondrial energy metabolism and antioxidant defense, enhanced ATP synthesis capacity by promoting NADH generation in the tricarboxylic acid (TCA) cycle upon its activity restoration. Simultaneously, its catalytic production of NADPH provided essential reducing equivalents for the glutathione (GSH) antioxidant system, significantly bolstering the cellular capacity to scavenge excess ROS (28, 29). H*if3a*, a negative regulator of the hypoxia-inducible factor family, antagonizes *Hif-1α* activity upon upregulation, shifting cellular metabolism from a glycolytic mode back to oxidative phosphorylation. This shift not only improves energy production efficiency but also reduces lactate accumulation and associated oxidative stress, indirectly supporting stable ATP synthesis and ROS level control (30).

During CA-CPR, ischemic–hypoxic mitochondrial injury exacerbates the production of ROS and the leakage of mitochondrial DNA (mtDNA), amplifying apoptotic cascades (31). Concurrently, deficiency in Epsa1 further promotes mitochondrial fission driven by *Drp1* and the release of mtDNA. In this pathological context, mitophagy—a selective autophagic process for clearing dysfunctional mitochondria orchestrated by *Pink1/Parkin*—emerges as a critical protective mechanism that maintains mitochondrial homeostasis and exerts anti-inflammatory effects (32–34). Heparin precisely targets this pathway by upregulating *Epsa1*, restoring *Idh2* activity, and stabilizing *Hif3a*, thereby enhancing *Pink1/Parkin*-mediated mitophagy to eliminate impaired mitochondria, suppress sustained inflammatory activation, and ultimately reduce neuronal apoptosis and cerebral edema.

Our findings confirmed this mechanistic cascade at molecular, tissue, and integrated functional levels. Heparin intervention significantly reversed the downregulation of *Epsa1*, *Idh2*, and *Hif3a* mRNA in the hippocampus following CA-CPR, with high concordance between qPCR and RNA-seq results ensuring reliability. This transcriptional shift directly translated into ultrastructural protection of mitochondria, as evidenced (by intact cristae and continuous outer membranes observed via electron microscopy). It also resulted in the restoration of energy metabolism, indicated by elevated ATP levels, and the mitigation of oxidative stress, as shown by reduced ROS levels. These microscopic enhancements culminated in macroscopic functional gains, including a significant reduction in neuronal apoptosis, a marked attenuation of cerebral edema, sustained improvement in neurobehavioral scores, and substantially increased 20-day survival rates.

In summary, we outline a clear causal chain: Heparin → upregulation of *Epsa1/Idh2/Hif3a* → Mitochondrial structural stabilization → Protection of mitochondrial function → Reduction of apoptosis and cerebral edema → Ultimate improvement of neurological function and survival outcome. This mechanism not only clarifies the pleiotropic neuroprotective effects of heparin beyond its anticoagulant role but also highlights the pivotal value of mitochondria-targeted therapeutic strategies in post-resuscitation brain injury.

Despite these mechanistic insights, our study has limitations: the relatively small sample size may constrain statistical power, and quantitative relationships between gene regulation and functional parameters remain to be established. Future studies should expand cohort sizes and directly validate the functional necessity of *Epsa1*, *Idh2*, and *Hif3a* through gene knockdown/overexpression experiments, thereby providing a robust experimental foundation for developing novel neuroprotective strategies.

In conclusion, this study, which employs a bioinformatics-driven experimental approach, is the first to systematically elucidate the molecular mechanism by which low-dose heparin exerts neuroprotection through multi-target mitochondrial regulation. It offers innovative concepts and theoretical underpinnings for the clinical management of brain injury following CA-CPR.

## 5 Conclusion

This study demonstrates that heparin treatment significantly improves neurological outcomes and survival rates in a rat model of cardiac arrest-cardiopulmonary resuscitation (CA-CPR), attenuating cerebral edema and preserving neuronal and mitochondrial integrity. Transcriptomic analysis revealed that heparin exerts neuroprotection through multi-pathway regulation, including modulation of the AGE-RAGE signaling and complement/coagulation cascades. We identified three mitochondrial-related core genes—*Epsa1*, *Idh2*, and *Hif3a*—as key mediators. Heparin upregulates *Epsa1* to inhibit DrP1-dependent mitochondrial fission, restores *Idh2* activity to enhance antioxidant capacity and reduce ROS, and stabilizes Hif3α to regulate glucose metabolism. Consequently, heparin maintains mitochondrial bioenergetics and structural homeostasis, thereby ameliorating oxidative stress and energy deficits. These findings provide transcriptomic-level evidence supporting heparin as a neuroprotective adjuvant via multi-target mitochondrial regulation, highlighting *Epsa1*, *Idh2*, and *Hif3a* as potential therapeutic targets for post-resuscitation brain injury.

## Data Availability

The original contributions presented in the study are included in the article/supplementary material, further inquiries can be directed to the corresponding authors.

## Footnotes

1
https://www.ncbi.nlm.nih.gov/gene
